# Supplementary material for: Estimation of the Circadian Phase Difference in Weekend Sleep and Further Evidence for Our Failure to Sleep More on Weekends to Catch Up on Lost Sleep
Source: Clocks Sleep. 2025 Nov 27;7(4):67. doi: 10.3390/clockssleep7040067 (PMC12731511; doi:10.3390/clockssleep7040067)
Supplement: Supplementary file 1 [file clockssleep-07-00067-s001.zip › clockssleep-3895593-supplementary.pdf]

Supplementary Materials

to

"Estimation of the Circadian Phase Difference in Weekend Sleep and Further Evidence for Our Failure to Sleep More on Weekends to Catch Up on Lost Sleep"

Supplementary Tables

Table S1. Distribution of survey participants into subsamples in accord with chronotype, age, sex, and weekday risetime.

	Age, yrs	17-25			>25			Whole sample		
	Type	Male	Female	Total	Male	Female	Total	Male	Female	Total
Weekday risetime (wRT) <7:00	Morning	90	254	344	15	83	98	105	337	442
	Evening	125	413	538	11	29	40	136	442	578
	Lethargic	66	204	270	10	37	47	76	241	317
	Vigilant	102	112	214	10	24	34	112	136	248
	Napping	68	264	332	9	39	48	77	303	380
	Afternoon	84	304	388	8	34	42	92	338	430
	Inconclusive	42	65	107	4	6	10	46	71	117
	Total	577	1616	2193	67	252	319	644	1868	2512
Weekday risetime (wRT) ≥7:00	Morning	95	179	274	8	32	40	103	211	314
	Evening	189	458	647	14	53	67	203	511	714
	Lethargic	68	165	233	7	38	45	75	203	278
	Vigilant	105	119	224	16	13	29	121	132	253
	Napping	73	194	267	11	31	42	84	225	309
	Afternoon	118	280	398	9	30	39	127	310	437
	Inconclusive	46	64	110	6	7	13	52	71	123
	Total	694	1459	2153	71	204	275	765	1663	2428
Total		1271	3075	4346	138	456	594	1409	3531	4940

Notes. Number of survey participants in subsamples with different ages, sexes, weekday risetimes (wRT), and chronotypes. Age 17-25 yrs (university student) and >25 yrs (lecturer or other staff member), Male and Female, before 7:00 and later, and one of 7 LIVEMAN types chosen among 7 options illustrating and shortly describing the patterns of daily change in alertness level (Morning: high in the morning, middle in the afternoon, and low in the evening; Evening: low in the morning, middle in the afternoon, and high in the evening; Lethargic: low in the morning, afternoon, and evening; Vigilant: high in the morning, afternoon, and evening; Napping: high in the morning, low in the afternoon, and middle in the evening; Afternoon: low in the morning, high in the afternoon, and middle in the evening; Inconclusive: none of the above).

Table S2. Four-, three-, and two-way ANOVAs of sleep times.

Four-way ANOVAs / Independent factor / Sleep time, its abbreviation			Main effect (four factors)				Some of interactions		
			1.Type	2.Age	3.Sex	4.wRT	1x4	2x4	1x2x3x4
			$F_{6/4884}$	$F_{1/4884}$	$F_{1/4884}$	$F_{1/4884}$	$F_{6/4884}$	$F_{1/4884}$	$F_{6/4884}$
Time in bed	Weekday	wTiB	7.9***	59.9***	0.9	132.3***	0.7	4.7*	0.9
	Weekend	fTiB	1.3	8.9**	1.3	3.6	0.6	0.0	0.6
	Gap	fwTiB	3.7**	69.5***	2.7	49.8***	0.6	2.8	1.7
Rise-time	Weekday	wRT	4.3***	0.0	4.4*	-	1.8	2.6	1.3
	Weekend	fRT	20.2***	122.6***	0.2	66.8***	1.7	0.5	2.1
	Gap	fwRT	12.7***	118.1***	0.4	77.5***	2.1	2.2	3.0**
Bed-time	Weekday	wBT	15.4***	62.7***	0.0	36.9***	1.2	2.1	0.7
	Weekend	fBT	18.3***	51.1***	2.3	31.1***	0.5	0.4	0.7
	Gap	fwBT	1.7	0.0	2.1	0.1	1.1	0.4	0.4

ANOVAs (main effects) / Sample/Age subsamples / Independent factor / Sleep time, its abbreviation			Three-way (three factors)			Two-way (two factors)			
			Whole			Age 17-25 yrs		Age >25 yrs	
			1.Type	2.Age	3.Sex	1.Type	2.Sex	1.Type	2.Sex
			$F_{6/4912}$	$F_{1/4912}$	$F_{1/4912}$	$F_{6/4332}$	$F_{1/4332}$	$F_{6/580}$	$F_{1/580}$
Time in bed	Weekday	wTiB	5.0***	51.8***	2.2	21.0***	15.7***	2.1	0.0
	Weekend	fTiB	1.3	9.2**	0.9	3.2**	12.0**	1.5	0.1
	Gap	fwTiB	2.4*	65.7***	3.8	17.5***	35.1***	0.7	0.0
Rise-time	Weekday	wRT	7.7***	0.0	7.9**	8.9***	15.8***	6.0***	3.3
	Weekend	fRT	23.4***	127.0***	0.7	61.7***	0.0	8.1***	1.0
	Gap	fwRT	8.9***	111.0***	1.1	36.1***	6.8**	2.5*	0.1
Bed-time	Weekday	wBT	19.2***	65.3***	0.2	44.2***	2.7	10.3***	1.8
	Weekend	fBT	21.9***	54.7***	3.1	40.1***	11.9**	14.6***	0.7
	Gap	fwBT	1.7	0.1	2.1	1.8	25.5***	2.5*	0.2

Notes. F-ratio from the results of four-, three-, and two-way ANOVAs of sleep times; $^{*}p < 0.05$, $^{**}p < 0.01$, $^{***}p < 0.001$ for F_{df}. The 4th factor was wRT (weekday risetime either before 7:00 or later). Some of interactions: Only two from all interactions were significant. See also other notes to Table 1, mean sleep times obtained by averaging within the whole sample and the age subsamples in Tables S3 and S4, the results of *post-hoc* pairwise comparison of these times in chronotypes in Table 2, see Tables S5-S16 for the results of *post-hoc* pairwise comparison of some of earlier and later risers, and see the illustration of mean times of chronotypes obtained by averaging within sex and age subsamples in Figures S1-S3.

Table S3. Times in bed and gaps in time in bed and risetime in the whole sample and age subsamples.

	Sleep time	Time in bed				Gap in			
		Weekday		Weekend		Time in bed		Risetime	
	Type	Mean	SEM	Mean	SEM	Mean	SEM	Mean	SEM
Whole sample	Morning	7.37	0.11	8.97	0.11	1.60	0.14	2.10	0.11
	Evening	6.64	0.11	8.82	0.11	2.18	0.13	3.05	0.11
	Lethargic	7.03	0.13	8.98	0.13	1.95	0.16	2.81	0.13
	Vigilant	7.12	0.12	8.71	0.13	1.59	0.15	2.19	0.12
	Napping	6.90	0.12	8.95	0.13	2.05	0.15	2.66	0.12
	Afternoon	7.29	0.13	9.17	0.13	1.88	0.16	2.72	0.13
Age17-25 yrs	Morning	7.24	0.08	8.92	0.08	1.68	0.10	2.35	0.08
	Evening	6.26	0.06	9.06	0.06	2.81	0.07	3.64	0.06
	Lethargic	6.78	0.09	9.32	0.09	2.55	0.11	3.46	0.09
	Vigilant	6.84	0.09	8.86	0.09	2.03	0.11	2.69	0.09
	Napping	6.38	0.09	9.17	0.09	2.79	0.11	3.40	0.09
	Afternoon	6.93	0.07	9.19	0.08	2.26	0.09	3.03	0.07
Age>25 yrs	Morning	7.50	0.20	9.02	0.21	1.52	0.26	1.85	0.17
	Evening	7.01	0.20	8.58	0.21	1.56	0.26	2.45	0.17
	Lethargic	7.28	0.24	8.63	0.25	1.36	0.30	2.15	0.20
	Vigilant	7.41	0.23	8.56	0.24	1.16	0.29	1.70	0.19
	Napping	7.42	0.22	8.73	0.23	1.31	0.29	1.91	0.19
	Afternoon	7.65	0.24	9.15	0.25	1.51	0.31	2.41	0.20

Notes. Mean and SEM: Mean time in bed or gap in weekend-weekday time in bed or risetime obtained by averaging within the whole sample or an age subsample and Standard Error of this Mean from three-way ANOVAs reported in Table S2. See also Table 1 for other notes and see Table 2 for the results of *post-hoc* pairwise comparison of types.

Table S4. Rise- and bedtime in the whole sample and age subsamples.

	Sleep time	Risetime				Bedtime			
		Weekday		Weekend		Weekday		Weekend	
	Type	Mean	SEM	Mean	SEM	Mean	SEM	Mean	SEM
Whole sample	Morning	6.67	0.07	8.77	0.10	23.30	0.10	23.81	0.11
	Evening	7.33	0.07	10.38	0.10	24.68	0.10	25.54	0.11
	Lethargic	6.82	0.09	9.63	0.12	23.80	0.11	24.65	0.13
	Vigilant	6.92	0.08	9.11	0.12	23.80	0.11	24.40	0.12
	Napping	6.96	0.08	9.62	0.12	24.06	0.11	24.67	0.12
	Afternoon	6.92	0.09	9.65	0.12	23.64	0.11	24.48	0.13
Age17-25 yrs	Morning	6.88	0.05	9.23	0.08	23.64	0.07	24.32	0.08
	Evening	7.23	0.04	10.88	0.06	24.94	0.05	25.77	0.06
	Lethargic	6.82	0.06	10.29	0.09	24.05	0.08	24.96	0.09
	Vigilant	6.91	0.06	9.60	0.08	24.08	0.08	24.74	0.09
	Napping	6.91	0.06	10.31	0.08	24.53	0.08	25.14	0.09
	Afternoon	6.99	0.05	10.02	0.07	24.06	0.07	24.85	0.08
Age >25 yrs	Morning	6.47	0.12	8.32	0.17	22.97	0.14	23.30	0.15
	Evening	7.43	0.12	9.88	0.17	24.42	0.14	25.31	0.15
	Lethargic	6.82	0.14	8.97	0.20	23.54	0.16	24.34	0.18
	Vigilant	6.93	0.13	8.62	0.19	23.52	0.16	24.06	0.17
	Napping	7.02	0.13	8.93	0.19	23.60	0.15	24.20	0.17
	Afternoon	6.86	0.14	9.27	0.20	23.21	0.17	24.12	0.18

Notes. Mean and SEM: Mean rise- or bedtime obtained by averaging within the whole sample or an age sample and Standard Error of this Mean from three-way ANOVAs reported in Table S2. See also other notes to Tables 1 and 2.

Table S5. Difference in sleep times between earlier and later weekday risers.

Difference, its abbreviation			Morning	Evening	Lethargic	Vigilant	Napping	Afternoon
Time in bed	Weekday	ΔwTiB	1.32***	1.42***	1.50***	1.56***	1.15***	1.08***
	Weekend	ΔfTiB	0.20	0.05	0.17	0.55	-0.04	0.13
	Gap	ΔfwTiB	-1.12***	-1.38***	-1.33***	-1.01***	-1.19***	-0.95***
Rise-time	Weekday	ΔwRT	1.70***	1.85***	1.70***	1.67***	1.53***	1.54***
	Weekend	ΔfRT	0.72***	0.57***	0.61**	0.90***	0.44*	0.54***
	Gap	ΔfwRT	-0.98***	-1.28***	-1.08***	-0.77***	-1.09***	-1.00***
Bed-time	Weekday	ΔwBT	0.38	0.40**	0.20	0.10	0.39	0.47**
	Weekend	ΔfBT	0.53**	0.49***	0.44	0.35	0.48*	0.42
	Gap	ΔfwBT	0.15	0.10	0.25	0.25	0.10	-0.05

Notes. The results of *post-hoc* pairwise comparison of sleep times in each of chronotypes that were further divided in accord with weekday risetime either before 7:00 or later (Table S1): $^{*}p < 0.05$, $^{**}p < 0.01$, $^{***}p < 0.001$ for t-score. The results support the predictions of non-significant difference in fTiB (ΔfTiB) between study participants despite the significant difference in weekday time in bed wTiB (ΔfTiB) and weekday risetime (ΔfwRT) that is the difference in weekday sleep loss. However, this difference in fwRT is partly compensated by the difference in weekend advance of the circadian sleep phase, i.e., the difference in fRT (ΔfRT), ΔwRT=ΔfRT+(-ΔfwRT). See also Table S2 for the results of preceding four-way ANOVAs, and see Figure 4A for illustration of difference between earlier and later risers in weekend sleep times.

Table S6. Difference in sleep times between evening and napping and between lethargic and afternoon types.

		Age, yrs	17-25				>25			
		Sex	Male		Female		Male		Female	
Difference, its abbreviation			Mean	SEM	Mean	SEM	Mean	SEM	Mean	SEM
Difference in sleep times between evening and napping types										
Time in bed	Weekday	ΔwTiB	-0.08	0.20	-0.17	0.10	-0.20	0.19	0.03	0.20
	Weekend	ΔfTiB	-0.03	0.20	-0.18	0.11	-0.02	0.19	-0.06	0.22
	Gap	ΔfwTiB	0.05	0.24	-0.01	0.13	0.18	0.22	-0.10	0.25
Rise-time	Weekday	ΔwRT	0.34	0.14	0.32***	0.07	0.31	0.13	0.66**	0.16
	Weekend	ΔfRT	0.62*	0.20	0.52***	0.09	0.72**	0.19	0.87**	0.22
	Gap	ΔfwRT	0.28	0.20	0.20	0.10	0.41	0.19	0.21	0.21
Bed-time	Weekday	ΔwBT	0.34	0.18	0.48***	0.09	0.45	0.17	0.63*	0.19
	Weekend	ΔfBT	0.57	0.20	0.70***	0.10	0.68**	0.19	0.93***	0.21
	Gap	ΔfwBT	0.23	0.19	0.21	0.10	0.23	0.17	0.31	0.17
Difference in sleep times between lethargic and afternoon types										
Time in bed	Weekday	ΔwTiB	-0.16	0.22	-0.14	0.12	-0.21	0.21	0.09	0.20
	Weekend	ΔfTiB	0.13	0.22	0.14	0.12	0.03	0.21	-0.16	0.23
	Gap	ΔfwTiB	0.29	0.26	0.28	0.16	0.24	0.24	-0.25	0.27
Rise-time	Weekday	ΔwRT	-0.27	0.15	-0.07	0.08	-0.27	0.14	0.15	0.16
	Weekend	ΔfRT	0.34	0.22	0.19	0.11	0.26	0.20	-0.36	0.23
	Gap	ΔfwRT	0.61	0.22	0.26	0.12	0.53	0.21	-0.51	0.22
Bed-time	Weekday	ΔwBT	-0.10	0.20	0.08	0.10	-0.05	0.19	0.06	0.20
	Weekend	ΔfBT	0.18	0.22	0.05	0.12	0.20	0.21	-0.20	0.22
	Gap	ΔfwBT	0.28	0.21	-0.03	0.12	0.25	0.19	-0.26	0.18

Notes Mean and SEM: Mean difference in sleep times between types obtained by averaging within male and female subsamples and Standard Error of this Mean from four-way ANOVAs. Significance of the difference between two types in sleep times with Bonferroni correction for the number of *post hoc* pairwise comparisons of 7 types: $^{*}p < 0.05$, $^{**}p < 0.01$, $^{***}p < 0.001$ for t-score. Since the results confirm the expectation that ΔfwRT does not differ from zero (Figure 3), ΔfRT is the estimate of the difference between these types in the circadian phase of sleep. See also other notes to Tables 1, 2, and S2-S5.

Table S7. Difference between morning and evening types in sleep times.

Age, yrs			17-25				>25			
Sex			Male		Female		Male		Female	
Difference, its abbreviation			Mean	SEM	Mean	SEM	Mean	SEM	Mean	SEM
Time in bed	Weekday	ΔwTiB	1.11***	0.19	0.86***	0.10	0.47	0.37	0.51	0.17
	Weekend	ΔfTiB	-0.32	0.19	0.03	0.11	0.94	0.57	-0.06	0.19
	Gap	ΔfwTiB	-1.42***	0.22	-0.83***	0.14	0.48	0.62	-0.56	0.23
Rise-time	Weekday	ΔwRT	-0.32	0.13	-0.40***	0.07	-0.95	0.35	-0.98***	0.14
	Weekend	ΔfRT	-1.86***	0.18	-1.44***	0.10	-1.42	0.56	-1.71***	0.19
	Gap	ΔfwRT	-1.53***	0.18	-1.04***	0.10	-0.47	0.60	-0.73**	0.19
Bed-time	Weekday	ΔwBT	-1.35***	0.17	-1.25***	0.09	-1.42*	0.40	-1.49***	0.17
	Weekend	ΔfBT	-1.45***	0.19	-1.47***	0.11	-2.36***	0.42	-1.65***	0.19
	Gap	ΔfwBT	-0.10	0.17	-0.22	0.11	-0.95	0.35	-0.16	0.15

Notes Mean and SEM: Mean difference in sleep times between morning and evening types obtained by averaging within male and female subsamples and Standard Error of this Mean from three-way ANOVAs. Significance of the difference between two types in sleep times with Bonferroni correction for the number of *post hoc* pairwise comparisons of 7 types: *$p < 0.05$, **$p < 0.01$, ***$p < 0.001$ for t-score. The results confirm the expectation (Figures 1-3) that ΔfRT≠0, contrary to the suggested in (A), ΔfwRT≠0, contrary to the suggested in (B), and, ΔfRT-ΔfwRT≠0, contrary to the suggested in (C), in the equation ΔwRT=ΔfRT+(-ΔfwRT). See also other notes to Tables 1, 2, and S2-S6.

Table S8. Difference in sleep times between earlier morning and later evening type risers.

Difference, its abbreviation			Subsample of the whole sample: Whole	Age, yrs: 17-25	Age, yrs: >25	Sex: Male	Sex: Female
Morning type with wRT<7:00 vs. evening type with wRT≥7:00							
Time in bed	Weekday	ΔwTiB	-0.24	-0.39	0.01	-0.13	-0.28
	Weekend	ΔfTiB	-0.18	-0.20	0.15	-0.40	-0.12
	Gap	ΔfwTiB	0.06	0.18	0.13	-0.27	0.16
Risetime	Weekday	ΔwRT	-1.98***	-1.97***	-2.07***	-2.12***	-1.92***
	Weekend	ΔfRT	-2.22***	-2.09***	-2.10***	-2.58***	-2.10***
	Gap	ΔfwRT	-0.24	-0.12	-0.03	-0.46	-0.18
Bedtime	Weekday	ΔwBT	-1.71***	-1.55***	-2.08***	-1.88***	-1.64***
	Weekend	ΔfBT	-2.01***	-1.85***	-2.24***	-2.07***	-1.98***
	Gap	ΔfwBT	-0.30	-0.31	-0.16	-0.19	-0.34
Morning type with wRT≥7:00 vs. evening type with wRT<7:00							
Time in bed	Weekday	ΔwTiB	2.50***	2.56***	1.54***	2.52***	2.49***
	Weekend	ΔfTiB	0.06	0.08	0.11	0.01	0.10
	Gap	ΔfwTiB	-2.44***	-2.48***	-1.43*	-2.51***	-2.38***
Risetime	Weekday	ΔwRT	1.57***	1.62***	1.17***	1.75***	1.46***
	Weekend	ΔfRT	-0.93***	-0.89***	-0.79	-1.00**	-0.95***
	Gap	ΔfwRT	-2.50***	-2.51***	-1.96***	-2.75***	-2.41***
Bedtime	Weekday	ΔwBT	-0.94***	-0.95***	-0.37	-0.77	-1.03***
	Weekend	ΔfBT	-0.99***	-0.96***	-0.90	-1.00**	-1.05***
	Gap	ΔfwBT	-0.06	-0.01	-0.53	-0.23	-0.02

Notes. The results of *post-hoc* pairwise comparison of sleep times in morning and evening types with weekday risetime before 7:00 and later, respectively (upper part) and with weekday risetime at 7:00 or later and earlier than 7:00, respectively (lower part): $^{*}p < 0.05$, $^{**}p < 0.01$, $^{***}p < 0.001$ for t-score. The results support the prediction of non-significant difference between these types in fTiB (ΔfTiB) despite significant difference in weekday sleep loss (ΔfwRT). It follows that, when the difference in sleep loss on weekdays that is the differences in fwRT (ΔfwRT) and fTiB (ΔfTiB) is non-significant (upper part but not lower part), the difference in the circadian sleep timing is equal to the difference in fRT/fBT (ΔfRT/ΔfBT). Therefore, when wRT and fTiB are similar in any two individuals with similar fTiB, the difference between them in the circadian phase of sleep (ΔfRT) can be calculated from reported sleep times. See also Table S1 for the distribution of the survey participants into types with earlier and later wRT, and Figures 4B illustrating the comparison shown in the upper part of the table.

Table S9. Difference between chronotypes in weekend-weekday gap in risetime.

	Type<br>wRT	Morning<br><7:00	Evening<br>≥7:00	Lethargic<br>≥7:00	Napping<br>≥7:00	Afternoon<br>≥7:00
Type						Age > 25 yrs
Morning	<7:00		-0.03	0.22	0.44	-0.11
Evening	≥7:00	-0.12		0.25	0.47	-0.08
Lethargic	≥7:00	0.14	0.26		0.21	-0.34
Napping	≥7:00	0.13	0.25	-0.01		-0.55
Afternoon	≥7:00	0.36^{*}	0.49^{***}	0.23	0.24	
		Age 17-25 yrs				
						Female
Morning	<7:00		-0.18	0.26	0.18	0.27
Evening	≥7:00	-0.46		0.44^{**}	0.36^{*}	0.45^{**}
Lethargic	≥7:00	-0.42	0.04		-0.08	0.01
Napping	≥7:00	-0.23	0.23	0.19		0.09
Afternoon	≥7:00	-0.08	0.38	0.34	0.15	
		Male				

Notes. *Post hoc* pairwise comparisons of weekend-weekday gaps in risetime in age and sex subsamples (below and above the diagonal, respectively). Significance of the difference between types in time in bed with Bonferroni correction for the number of *post hoc* pairwise comparisons: $^{*}p < 0.05$, $^{***}p < 0.001$ for t-score. Sub-subsample of morning types with weekday risetime (wRT) earlier than 7:00 and subsamples of 4 other types with later weekday risetime were included in these comparisons. Vigilant type was not included in this comparison due to significantly shorter weekend time in bed than in other types. When the results confirm the expectation that difference in weekend-weekday gap in risetime is not significantly different from zero (Figure 3), the difference in weekend risetime is close to the difference between these types in the circadian sleep phase. See also Table S1 for the distribution of the survey participants into types and risers, Table S3 (upper part) for sample-averaged times obtained by averaging within the whole sample,

Table S10. Difference between chronotypes in weekend-weekday gap in time in bed.

	Type<br>wRT	Morning<br><7:00	Evening<br>≥7:00	Lethargic<br>≥7:00	Napping<br>≥7:00	Afternoon<br>≥7:00
Type						Age > 25 yrs
Morning	<7:00		0.13	0.79	0.45	0.28
Evening	≥7:00	0.18		0.65	0.32	0.15
Lethargic	≥7:00	0.46	0.28		-0.33	-0.51
Napping	≥7:00	0.21	0.02	-0.25		-0.18
Afternoon	≥7:00	0.50*	0.31	0.04	0.29	
		Age 17-25 yrs				
						Female
Morning	<7:00		0.16	0.51	0.29	0.44
Evening	≥7:00	-0.27		0.35	0.14	0.29
Lethargic	≥7:00	0.26	0.53		-0.21	-0.06
Napping	≥7:00	-0.26	0.01	-0.52		0.15
Afternoon	≥7:00	0.04	0.31	-0.22	0.30	
		Male				

Notes. *Post hoc* pairwise comparisons of weekend-weekday gaps in time in bed in age and sex subsamples (below and above the diagonal, respectively). Significance of the difference between types in time in bed with Bonferroni correction for the number of *post hoc* pairwise comparisons: $^{*}p < 0.05$, $^{***}p < 0.001$ for t-score. See also Table S9 for other notes, and Table S3 (upper part) for sample-averaged times obtained by averaging within the whole sample.

Table S11. Difference between chronotypes in weekend bedtime.

	Type wRT	Morning <7:00	Evening ≥7:00	Lethargic ≥7:00	Napping ≥7:00	Afternoon ≥7:00
Type						Age > 25 yrs
Morning	<7:00		-2.24***	-1.47***	-1.26***	-1.27***
Evening	≥7:00	-1.85***		0.77*	0.98**	0.97**
Lethargic	≥7:00	-1.03***	0.82***		0.21	0.19
Napping	≥7:00	-1.22***	0.63***	-0.19		-0.01
Afternoon	≥7:00	-0.95***	0.91***	0.08	0.28	
		Age 17-25 yrs				
						Female
Morning	<7:00		-1.98***	-0.98**	-1.24***	-1.11***
Evening	≥7:00	-2.07***		1.00**	0.74***	0.87***
Lethargic	≥7:00	-1.61***	0.45		-0.25	-0.13
Napping	≥7:00	-1.49***	0.58	0.13		0.12
Afternoon	≥7:00	-1.05**	1.02***	0.56	0.44	
		Male				

Notes. *Post hoc* pairwise comparisons of weekend bedtime in bed in age and sex subsamples (below and above the diagonal, respectively). Significance of the difference between types in time in bed with Bonferroni correction for the number of *post hoc* pairwise comparisons: $^{*}p < 0.05$, $^{***}p < 0.001$ for t-score. See also Table S9 for other notes, Table S3 (lower part) for sample-averaged times obtained by averaging within the whole sample, and Figure 5 illustrating these bedtimes in age subsamples.

Table S12. Difference between chronotypes in weekend risetime.

	Type<br>wRT	Morning<br><7:00	Evening<br>≥7:00	Lethargic<br>≥7:00	Napping<br>≥7:00	Afternoon<br>≥7:00
Type						Age > 25 yrs
Morning	<7:00		-2.10***	-1.50***	-1.18***	-1.61***
Evening	≥7:00	-2.09***		0.60	0.92*	0.49
Lethargic	≥7:00	-1.58***	0.52***		0.32	-0.11
Napping	≥7:00	-1.53***	0.56***	0.04		-0.43
Afternoon	≥7:00	-1.32***	0.77***	0.26	0.21	
		Age 17-25 yrs				
						Female
Morning	<7:00		-2.10***	-1.44***	-1.40***	-1.42***
Evening	≥7:00	-2.58***		0.66***	0.70***	0.69***
Lethargic	≥7:00	-2.16***	0.42		0.04	0.02
Napping	≥7:00	-2.06***	0.52	0.10		-0.01
Afternoon	≥7:00	-1.70***	0.88***	0.46	0.36	
		Male				

Notes. *Post hoc* pairwise comparisons of weekend risetime in age and sex subsamples (below and above the diagonal, respectively). Significance of the difference between types in time in bed with Bonferroni correction for the number of *post hoc* pairwise comparisons: $^{*}p < 0.05$, $^{***}p < 0.001$ for t-score. See also Table S9 for other notes, Table S3 (lower part) for sample-averaged times obtained by averaging within the whole sample, and Figure 5 illustrating these risetimes in age subsamples.

Table S13. Difference between chronotypes with either earlier or later weekday risetimes.

	Type<br>wRT	Morning<br><7:00	Evening<br>≥7:00	Lethargic<br>≥7:00	Napping<br>≥7:00	Afternoon<br>≥7:00
Type		Difference in weekend-weekday gap in time in bed				
Morning	<7:00		0.06	0.46	0.16	0.35
Evening	≥7:00	-0.24		0.40	0.10	0.29
Lethargic	≥7:00	0.09	0.33		-0.30	-0.10
Napping	≥7:00	0.08	0.32*	-0.01		0.19
Afternoon	≥7:00	0.19	0.43***	0.10	0.11	
		Difference in weekend-weekday gap in risetime				
		Difference in risetime on weekend				
Morning	<7:00		-2.22***	-1.63***	-1.57***	-1.48***
Evening	≥7:00	-2.01***		0.60***	0.65***	0.74***
Lethargic	≥7:00	-1.16***	0.85***		0.05	0.14
Napping	≥7:00	-1.31***	0.70***	-0.15		0.09
Afternoon	≥7:00	-1.10***	0.91***	0.06	0.21	
		Difference in bedtime on weekend				

Notes. *Post hoc* pairwise comparisons of weekend-weekday gaps in risetime and times in bed (upper part, below and above the diagonal, respectively) and weekend rise- and bedtime (lower part, above and below the diagonal, respectively). Significance of the difference between types with Bonferroni correction for the number of *post hoc* pairwise comparisons: $^{*}p < 0.05$, $^{***}p < 0.001$ for t-score. Sub-subsample of morning types with weekday risetime (wRT) earlier than 7:00 and Subsamples of 4 other types with later weekday risetime were compared. Vigilant type was not included in this comparison due to a significantly shorter weekend time in bed compared to other types. When the results confirm the expectation that difference in gap in risetime is not significantly different from zero (see the legend to Figure 3), the difference in weekend risetime can be used as the estimate of the difference between these types in the circadian phase of sleep. See also Table S1 for the distribution of the survey participants into types and weekday risers, Table S3 (upper part) for sample-averaged times obtained by averaging within the whole sample, and Tables S9-S13 for the differences between sample- and subsample-averaged times.

Table S14. Sleep times in chronotypes with earlier and later weekday risetimes.

Sleep time, abbreviation		Type	Morning		Evening		Lethargic		Vigilant		Napping		Afternoon	
			Mean	SEM	Mean	SEM	Mean	SEM	Mean	SEM	Mean	SEM	Mean	SEM
Chronotype: Morning or Another (Evening, Lethargic, Vigilant, Napping, Afternoon)														
Time in bed	Weekday	wTiB	7.49	0.11	6.56	0.10	7.05	0.12	7.11	0.12	6.91	0.11	7.28	0.11
	Weekend	fTiB	8.96	0.12	8.82	0.11	8.99	0.14	8.71	0.13	8.96	0.12	9.16	0.12
	Gap	fwTiB	1.47	0.14	2.27	0.13	1.95	0.16	1.60	0.14	2.04	0.15	1.89	0.14
Rise-time	Weekday	wRT	6.85	0.06	7.18	0.05	6.86	0.06	6.94	0.06	6.98	0.05	6.90	0.06
	Weekend	fRT	8.84	0.11	10.33	0.10	9.67	0.13	9.13	0.11	9.65	0.11	9.63	0.11
	Gap	fwRT	1.99	0.11	3.15	0.10	2.81	0.13	2.19	0.11	2.67	0.11	2.73	0.11
Bed-time	Weekday	wBT	23.36	0.10	24.61	0.10	23.82	0.12	23.83	0.11	24.07	0.11	23.63	0.11
	Weekend	fBT	23.88	0.12	25.50	0.11	24.68	0.13	24.42	0.11	24.69	0.11	24.47	0.11
	Gap	fwBT	0.52	0.11	0.89	0.11	0.86	0.13	0.59	0.10	0.62	0.12	0.85	0.10
Earlier risers: Morning or Morning and Another (weekday risetime before 7:00), <7														
Time in bed	Weekday	wTiB	6.88	0.13	6.47	0.10	6.67	0.10	6.64	0.10	6.62	0.10	6.86	0.10
	Weekend	fTiB	8.92	0.14	8.87	0.11	8.89	0.12	8.70	0.11	8.96	0.11	8.99	0.11
	Gap	fwTiB	2.04	0.17	2.40	0.13	2.22	0.13	2.06	0.12	2.34	0.13	2.13	0.12
Rise-time	Weekday	wRT	5.99	0.07	6.13	0.06	6.01	0.05	6.09	0.05	6.09	0.05	6.10	0.05
	Weekend	fRT	8.49	0.13	9.28	0.10	8.80	0.11	8.59	0.10	9.00	0.10	8.88	0.10
	Gap	fwRT	2.50	0.13	3.15	0.10	2.79	0.11	2.51	0.10	2.92	0.10	2.78	0.10
Bed-time	Weekday	wBT	23.11	0.12	23.66	0.09	23.34	0.10	23.45	0.10	23.47	0.10	23.24	0.10
	Weekend	fBT	23.57	0.14	24.41	0.10	23.91	0.11	23.90	0.10	24.04	0.10	23.90	0.10
	Gap	fwBT	0.46	0.13	0.75	0.10	0.57	0.11	0.45	0.09	0.57	0.11	0.66	0.09
Later risers: Morning or Morning and Another (weekday risetime at 7:00 or later), ≥7														
Time in bed	Weekday	wTiB	8.10	0.17	7.58	0.11	7.87	0.13	7.96	0.12	7.78	0.11	7.90	0.11
	Weekend	fTiB	9.00	0.19	8.91	0.12	9.06	0.14	8.97	0.13	8.95	0.13	9.14	0.12
	Gap	fwTiB	0.90	0.23	1.34	0.14	1.19	0.16	1.02	0.14	1.17	0.15	1.23	0.14
Rise-time	Weekday	wRT	7.71	0.09	7.90	0.06	7.70	0.06	7.70	0.06	7.74	0.05	7.65	0.06
	Weekend	fRT	9.19	0.17	9.89	0.11	9.71	0.13	9.37	0.12	9.48	0.11	9.59	0.11
	Gap	fwRT	1.48	0.18	1.99	0.11	2.01	0.13	1.67	0.12	1.74	0.11	1.94	0.11
Bed-time	Weekday	wBT	23.61	0.16	24.31	0.10	23.84	0.12	23.75	0.11	23.96	0.11	23.75	0.11
	Weekend	fBT	24.20	0.19	24.97	0.11	24.65	0.14	24.40	0.11	24.53	0.12	24.46	0.11
	Gap	fwBT	0.59	0.18	0.66	0.10	0.82	0.13	0.66	0.10	0.57	0.12	0.71	0.10
Morning earlier risers or Another later risers, Morning<7 and Another≥7														
Time in bed	Weekday	wTiB	6.88	0.13	7.05	0.13	7.63	0.18	7.81	0.16	7.46	0.16	7.71	0.16
	Weekend	fTiB	8.92	0.14	8.83	0.14	9.13	0.20	8.95	0.18	8.91	0.17	9.27	0.18
	Gap	fwTiB	2.04	0.17	1.77	0.17	1.49	0.24	1.14	0.22	1.45	0.21	1.57	0.22
Rise-time	Weekday	wRT	5.99	0.07	8.09	0.07	7.69	0.09	7.69	0.08	7.77	0.08	7.59	0.08
	Weekend	fRT	8.49	0.13	10.60	0.13	10.23	0.18	9.56	0.16	9.78	0.16	9.98	0.16
	Gap	fwRT	2.50	0.13	2.51	0.13	2.55	0.19	1.87	0.17	2.00	0.16	2.39	0.17
Bed-time	Weekday	wBT	23.11	0.12	25.01	0.12	24.06	0.17	23.88	0.16	24.31	0.15	23.89	0.16
	Weekend	fBT	23.57	0.14	25.74	0.14	25.11	0.20	24.61	0.18	24.87	0.17	24.72	0.18
	Gap	fwBT	0.46	0.13	0.73	0.14	1.05	0.19	0.73	0.17	0.56	0.16	0.84	0.17

Note. These times are illustrated in Figure 4 and used to calculate the differences in sleep times reported in Table S16. From the results of ANOVAs of morning type and one of other types (Another).

Table S15. Sleep times in chronotypes with earlier and later weekday risetimes in younger and older ages.

Sleep time, abbreviation		Type	Morning		Evening		Lethargic		Vigilant		Napping		Afternoon	
			Mean	SEM	Mean	SEM	Mean	SEM	Mean	SEM	Mean	SEM	Mean	SEM
Chronotype in younger age: Morning or Another (Evening, Lethargic, Vigilant, Napping, Afternoon)														
Time in bed	Weekday	wTiB	7.30	0.07	6.17	0.06	6.81	0.09	6.82	0.08	6.42	0.08	6.90	0.06
	Weekend	fTiB	8.92	0.08	9.06	0.06	9.33	0.10	8.86	0.09	9.16	0.09	9.17	0.07
	Gap	fwTiB	1.63	0.09	2.90	0.07	2.52	0.11	2.04	0.09	2.74	0.10	2.26	0.08
Rise-time	Weekday	wRT	6.93	0.04	7.11	0.03	6.86	0.04	6.89	0.04	6.95	0.04	6.94	0.03
	Weekend	fRT	9.24	0.07	10.84	0.06	10.29	0.09	9.59	0.08	10.31	0.08	9.99	0.06
	Gap	fwRT	2.31	0.08	3.73	0.06	3.44	0.09	2.70	0.08	3.36	0.08	3.05	0.07
Bed-time	Weekday	wBT	23.64	0.07	24.92	0.05	24.05	0.08	24.08	0.08	24.53	0.08	24.04	0.06
	Weekend	fBT	24.32	0.08	25.75	0.06	24.96	0.09	24.73	0.08	25.15	0.08	24.84	0.06
	Gap	fwBT	0.68	0.07	0.84	0.05	0.91	0.09	0.66	0.07	0.62	0.08	0.80	0.06
Morning earlier younger risers or Another later younger risers, Morning<7 and Another≥7														
Time in bed	Weekday	wTiB	6.59	0.10	6.89	0.07	7.55	0.12	7.64	0.11	6.98	0.11	7.41	0.09
	Weekend	fTiB	8.76	0.11	9.09	0.08	9.37	0.14	9.15	0.12	9.25	0.13	9.29	0.09
	Gap	fwTiB	2.18	0.13	2.20	0.09	1.82	0.16	1.50	0.13	2.27	0.15	1.88	0.11
Rise-time	Weekday	wRT	6.05	0.06	8.05	0.04	7.76	0.06	7.75	0.05	7.73	0.05	7.71	0.04
	Weekend	fRT	8.85	0.10	11.11	0.07	10.61	0.13	10.05	0.11	10.65	0.11	10.30	0.08
	Gap	fwRT	2.81	0.11	3.07	0.07	2.85	0.13	2.30	0.11	2.92	0.11	2.59	0.09
Bed-time	Weekday	wBT	23.46	0.10	25.09	0.07	24.23	0.11	24.10	0.11	24.75	0.11	24.30	0.08
	Weekend	fBT	24.09	0.10	25.96	0.07	25.24	0.13	24.90	0.11	25.40	0.12	25.03	0.08
	Gap	fwBT	0.63	0.10	0.87	0.07	1.01	0.13	0.80	0.10	0.65	0.12	0.73	0.08
Chronotype in older ages: Morning or Another (Evening, Lethargic, Vigilant, Napping, Afternoon)														
Time in bed	Weekday	wTiB	7.68	0.20	6.95	0.19	7.28	0.23	7.40	0.22	7.41	0.21	7.65	0.21
	Weekend	fTiB	8.99	0.22	8.58	0.21	8.66	0.26	8.57	0.24	8.76	0.23	9.16	0.22
	Gap	fwTiB	1.31	0.25	1.63	0.24	1.38	0.30	1.17	0.26	1.35	0.27	1.51	0.26
Rise-time	Weekday	wRT	6.77	0.11	7.25	0.11	6.87	0.11	6.98	0.11	7.01	0.10	6.86	0.11
	Weekend	fRT	8.44	0.20	9.82	0.19	9.05	0.24	8.67	0.21	8.98	0.20	9.27	0.21
	Gap	fwRT	1.67	0.21	2.57	0.20	2.19	0.24	1.68	0.21	1.98	0.21	2.41	0.22
Bed-time	Weekday	wBT	23.09	0.19	24.30	0.18	23.59	0.22	23.58	0.21	23.60	0.20	23.22	0.20
	Weekend	fBT	23.45	0.21	25.24	0.20	24.40	0.25	24.10	0.21	24.23	0.21	24.11	0.21
	Gap	fwBT	0.36	0.19	0.94	0.18	0.81	0.24	0.52	0.19	0.63	0.21	0.90	0.20
Morning earlier older risers or Another later older risers, Morning<7 and Another≥7														
Time in bed	Weekday	wTiB	7.17	0.23	7.21	0.25	7.72	0.34	7.98	0.30	7.94	0.29	8.01	0.30
	Weekend	fTiB	9.08	0.25	8.56	0.27	8.88	0.39	8.76	0.34	8.56	0.32	9.26	0.31
	Gap	fwTiB	1.91	0.29	1.35	0.31	1.16	0.45	0.77	0.37	0.63	0.38	1.25	0.36
Rise-time	Weekday	wRT	5.93	0.13	8.14	0.14	7.61	0.17	7.64	0.15	7.82	0.13	7.48	0.15
	Weekend	fRT	8.12	0.23	10.08	0.25	9.85	0.36	9.07	0.30	8.91	0.28	9.67	0.29
	Gap	fwRT	2.19	0.24	1.94	0.26	2.24	0.36	1.43	0.30	1.09	0.28	2.19	0.30
Bed-time	Weekday	wBT	22.77	0.22	24.92	0.23	23.89	0.33	23.65	0.29	23.88	0.27	23.47	0.28
	Weekend	fBT	23.05	0.24	25.52	0.26	24.97	0.37	24.31	0.30	24.34	0.29	24.42	0.29
	Gap	fwBT	0.28	0.22	0.59	0.24	1.08	0.36	0.66	0.27	0.46	0.30	0.94	0.27

Note. These times are illustrated in Figure 5. See also Table 3 and other notes to Table S14.

Table S16. Difference in sleep times between morning and each of other chronotypes.

		Another type	Evening	Lethargic	Vigilant	Napping	Afternoon
Difference between Another and Morning type, all Another minus all Morning (uncorrected for risetime)							
Time	Weekday	ΔwTiB	-0.93***	-0.44**	-0.38*	-0.58***	-0.21
in	Weekend	ΔfTiB	-0.14	0.04	-0.25	0.00	0.20
bed	Gap	ΔfwTiB	0.80***	0.48*	0.14	0.57**	0.42*
Rise-	Weekday	ΔwRT	0.33***	0.01	0.09	0.13	0.05
time	Weekend	ΔfRT	1.49***	0.83***	0.29	0.81***	0.79***
	Gap	ΔfwRT	1.17***	0.82***	0.20	0.68***	0.74***
Bed-	Weekday	ΔwBT	1.25***	0.46**	0.47**	0.70***	0.27
time	Weekend	ΔfBT	1.61***	0.80***	0.53**	0.81***	0.59***
	Gap	ΔfwBT	0.37**	0.34*	0.06	0.10	0.33*
Difference between all later and all earlier weekday risers of Morning and Another types, ≥7 minus <7							
Time	Weekday	ΔwTiB	1.11***	1.20***	1.32***	1.16***	1.05***
in	Weekend	ΔfTiB	0.05	0.17	0.28	-0.01	0.15
bed	Gap	ΔfwTiB	-1.07***	-1.03***	-1.04***	-1.17***	-0.90***
Rise-	Weekday	ΔwRT	1.78***	1.69***	1.62***	1.66***	1.55***
time	Weekend	ΔfRT	0.62***	0.91***	0.78***	0.48**	0.70***
	Gap	ΔfwRT	-1.16***	-0.78***	-0.84***	-1.18***	-0.85***
Bed-	Weekday	ΔwBT	0.65***	0.49**	0.30*	0.50**	0.51***
time	Weekend	ΔfBT	0.56***	0.74***	0.51**	0.50**	0.56***
	Gap	ΔfwBT	-0.09	0.25	0.21	0.00	0.06
Difference between Another≥7 and Morning<7 (corrected for risetime)							
Time	Weekday	ΔwTiB	0.18	0.76*	0.94***	0.58	0.83*
in	Weekend	ΔfTiB	-0.09	0.21	0.03	-0.01	0.35
bed	Gap	ΔfwTiB	-0.27	-0.55	-0.91	-0.60	-0.48
Rise-	Weekday	ΔwRT	2.10***	1.70***	1.70***	1.79***	1.60***
time	Weekend	ΔfRT	2.11***	1.74***	1.07***	1.29***	1.50***
	Gap	ΔfwRT	0.01	0.05	-0.63	-0.50	-0.11
Bed-	Weekday	ΔwBT	1.89***	0.95**	0.77*	1.20***	0.77**
time	Weekend	ΔfBT	2.17***	1.54***	1.04***	1.30***	1.15***
	Gap	ΔfwBT	0.28	0.59	0.27	0.10	0.38

Notes. Results of ANOVAs provided the estimates of differences between sleep times of Morning types and each of other types (Table S14), Evening, Lethargic, Vigilant, Napping, Afternoon (Another). Upper part: Difference in sleep times between Another type and Morning type before correction for their difference in weekend-weekday gap in risetime (Another minus Morning). Middle part: …between earlier and later weekday risers (either earlier than 7:00 or later, ≥7 minus <7, in an analyzed sample consisting of morning and one of other types. Lower part: …between earlier weekday risers of morning type and later weekday risers of another type (Another≥7 minus Morning<7), i.e., the correction for difference in weekend-weekday gap in risetime that gives the difference between the circadian phases of sleep in this pair of (Morning and Another) types. Difference of lower part can be directly calculated as the sum of upper part and middle part differences: ≥7-<7+Another-Morning=(Morning≥7+Another≥7-Morning<7-Another<7)/2+(Another<7+Another≥7-Morning<7-Morning≥7)/2=Another≥7-Morning<7.

Table S17. ANOVAs of scores on scales assessing wakeability, sleepability and sleepiness.

Time of the day	Main effect of independent factor in ANOVAs<br>Questionnaire, scale, its abbreviation			Type $F_{6/4912}$	Sex $F_{1/4912}$	Age $F_{6/4912}$
Morning	VJT	Morning Sleepiness	SM	35.1***	5.2*	23.0***
Evening/night	VJT	Evening/night Sleepiness	SE	26.0***	53.6***	47.5***
Both	VJT	Difference between them	SM-E	49.7***	12.9***	71.0***
Morning	SWAT	Morning Sleepability	MS	51.4***	31.6***	35.3***
Evening/night	SWAT	Nighttime Wakeability	NW	80.0***	36.8***	60.2***
Both	SWAT	Their sum	NW+MS	98.4***	0.0	82.9***
Daytime	VJT	Daytime Sleepiness	SD	10.9***	19.3***	5.8*
Daytime	SWAT	Daytime Wakeability	DW	44.8***	13.8***	1.7
Daytime	SWAT	Daytime Sleepability	DS	6.7***	8.8**	12.4***

Notes. F-ratio from the results of three-way ANOVAs of scores on questionnaire scales designed to assess either phase of daily rhythm (alertness-sleepiness levels before and after sleep, upper and middle part) or daytime level of alertness-sleepiness (lower part); $^{*}p < 0.05$, $^{**}p < 0.01$, $^{***}p < 0.001$ for F_{df}. SWAT: Scores on 5 10-item scales (for any scale, a positive score denotes lateness or ability either to sleep or to remain awake at certain time of the day); VJT: Scores on the Karolinska Sleepiness Scale (KSS) averaged within three intervals of 1.5-day wakefulness (a higher score denotes a higher sleepiness level). Mean scores obtained by averaging within sex and age subsamples for LIVEMAN types are illustrated in Figures S5-S7.

Captions to Supplementary Figures

Figure S1. Reported times in bed.

Time in bed on weekdays (A) and weekends (B), and weekend-weekday gap in the time (C). Times are given in hours, but shown relative to the sample-averaged times obtained in ANOVAs (Table S2). Times averaged over sexes and over sexes x ages are reported in Table S3, and the results of post hoc pairwise comparison of chronotypes are reported in Table 2.

Figure S2. Reported risetimes.

Risetime on weekdays (A) and weekends (B), and weekend-weekday gap in risetime (C). Times are given in clock hours, but shown relative to the sample-averaged clock times obtained in ANOVAs (Table S2). See also the results of post hoc pairwise comparison of chronotypes in Table 2 and the caption to Figure S1.

Figure S3. Reported bedtimes.

Bedtime on weekdays (A) and weekends (B), and weekend-weekday gap in bedtime (C). See also captions to Figures S1 and S2.

Figure S4. Shift of weekend bed- and risetimes in response to earlier weekday risetime.

(A)-(B) Chronotype-averaged mean weekend bed- and risetimes (fBT and fRT, respectively) and their Standard Errors from the four-way ANOVAs (Table S2) with the 4th independent factor "Weekday risetime" (either before 7:00 or later). (A): Averaged over age and sex subsamples; Age 17-25 yrs and >25 years (B and C): Averaged within each of two age subsamples and over sexs; Male and Female (D and E): Averaged within each of two sexes and over ages. See also captions to Figures S1-S3.

Figure S5. The 1.5-day time course of expected sleepiness.

(A)-(C). Comparison of 6 chronotypes chosen from 7 chart options illustrating and shortly describing the patterns of daily change in alertness level (Morning: high in the morning, middle in the afternoon, and low in the evening; Evening: low in the morning, middle in the afternoon, and high in the evening; Lethargic: low in the morning, afternoon, and evening; Vigilant: high in the morning, afternoon, and evening; Napping: high in the morning, low in the afternoon, and middle in the evening; Afternoon: low in the morning, high in the afternoon, and middle in the evening). Scores on the Karolinska Sleepiness Scale (KSS) on the interval of 1.5-day wakefulness from the VJT (i.e., a higher score denotes a higher sleepiness level).

Figure S6. Sleepiness expected in the morning and evening/night hours.

(A)-(C). Scores on the Karolinska Sleepiness Scale (KSS) averaged within two subintervals of wakefulness (i.e., a higher score denotes a higher sleepiness level). Scores are shown relative to the sample-averaged score obtained in three-way ANOVAs (Table S17). See also the caption to Figures S1-S5.

Figure S7. Morning sleepability and evening/night wakeability.

(A)-(C). Scores on two 10-item scales of the SWAT (for any scale and for difference between the scales, a positive score denotes lateness). See also the caption to Figures S1-S6.

Figure S4. Daytime wakeability, sleepability, and sleepiness.

(A) and (B). Scores on two 10-item scales of the SWAT (for any scale, a positive score denotes ability either to sleep or to remain awake at certain time of the day). (C) Scores on the Karolinska Sleepiness Scale (KSS) averaged within daytime interval of 1.5-day wakefulness (i.e., a higher score denotes a higher sleepiness level). See also captions to Figures S1-S7.

Supplementary Figure

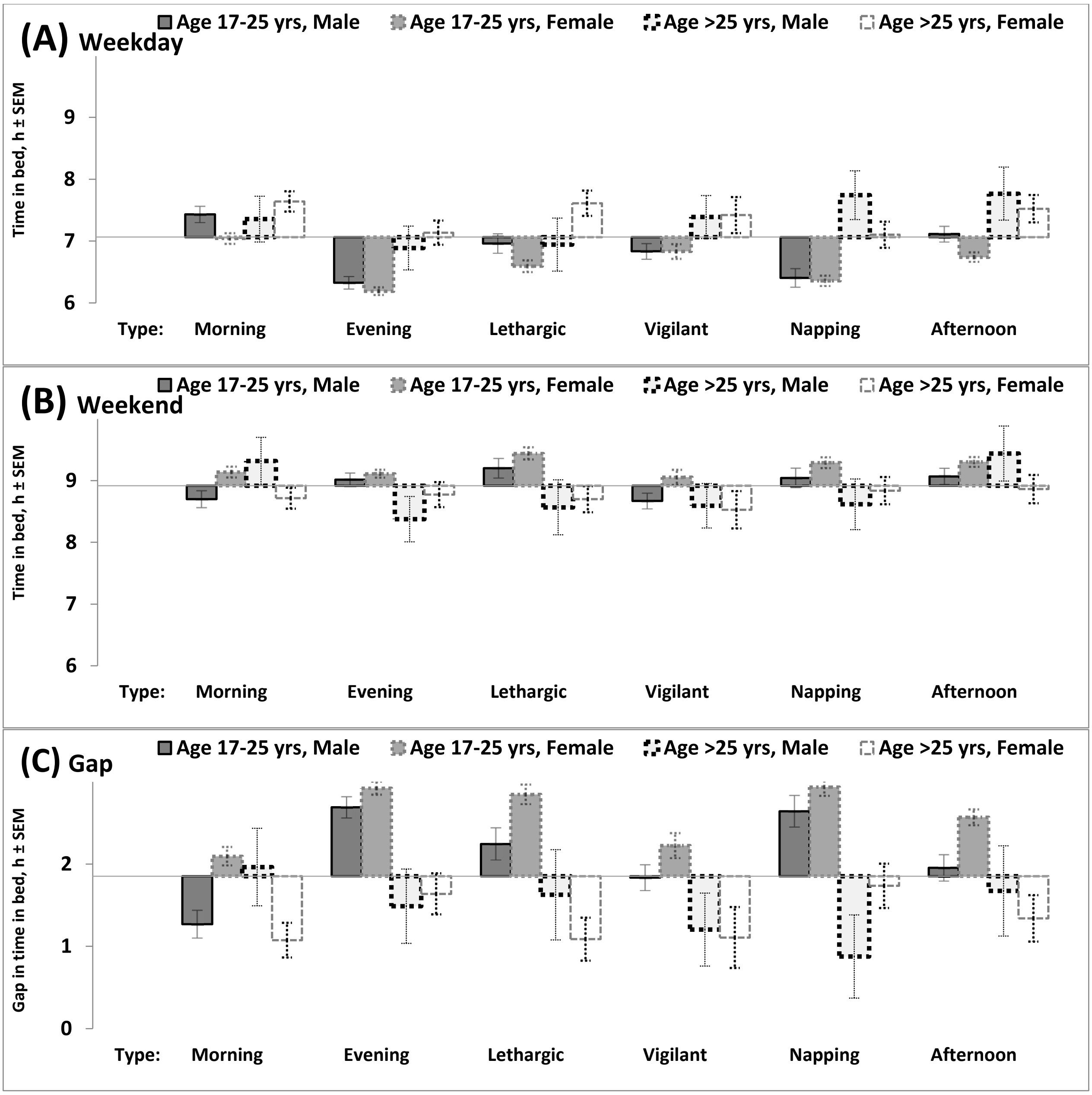

Figure S1

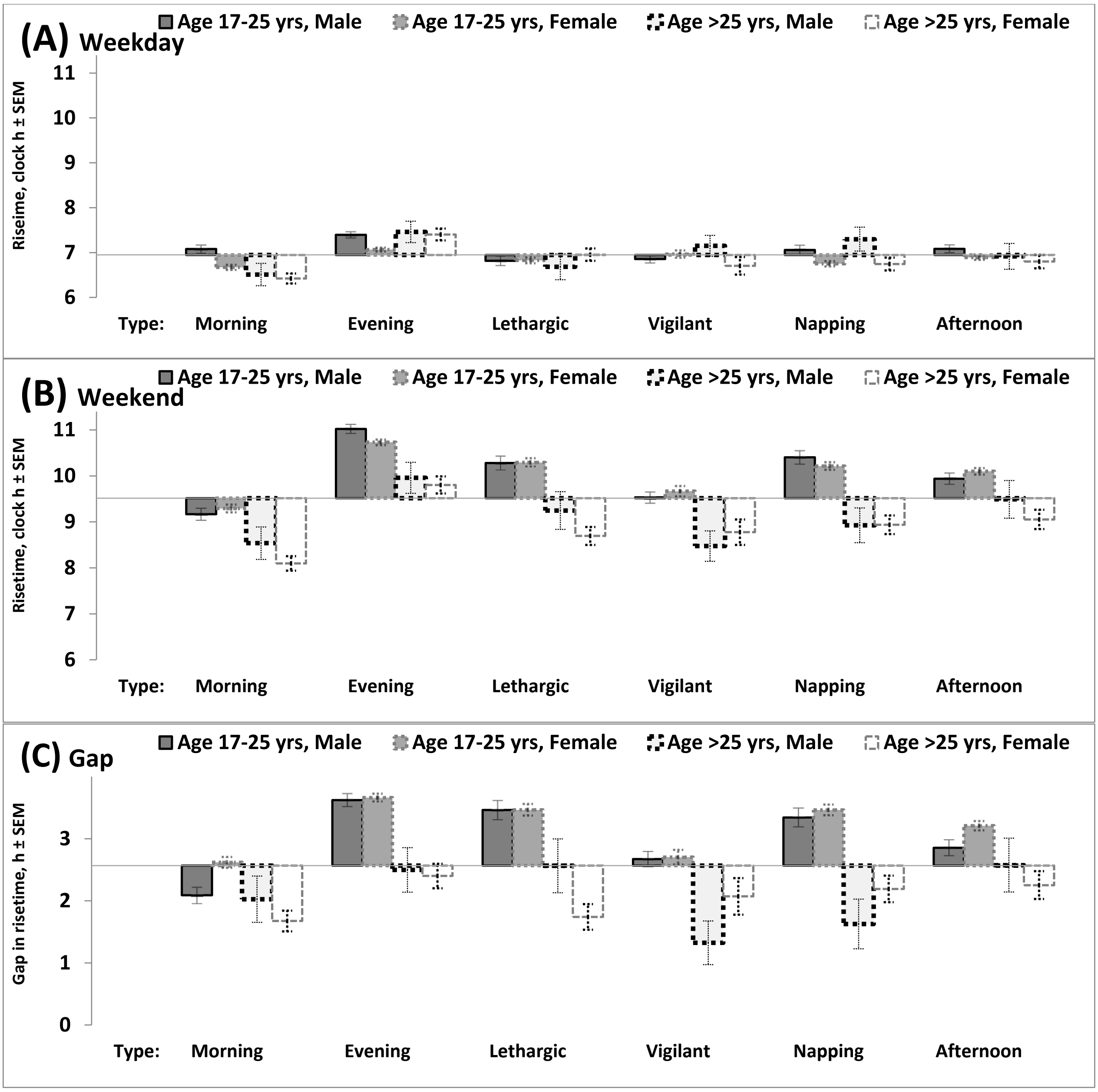

(A) Weekday
Age 17-25 yrs, Male
Age 17-25 yrs, Female
Age >25 yrs, Male
Age >25 yrs, Female
Riseime, clock h ± SEM
11
10
9
8
7
6
Type:
Morning
Evening
Lethargic
Vigilant
Napping
Afternoon
(B) Weekend
Risetime, clock h ± SEM
(C) Gap
Gap in risetime, h ± SEM
3
2
1
0

Figure S2

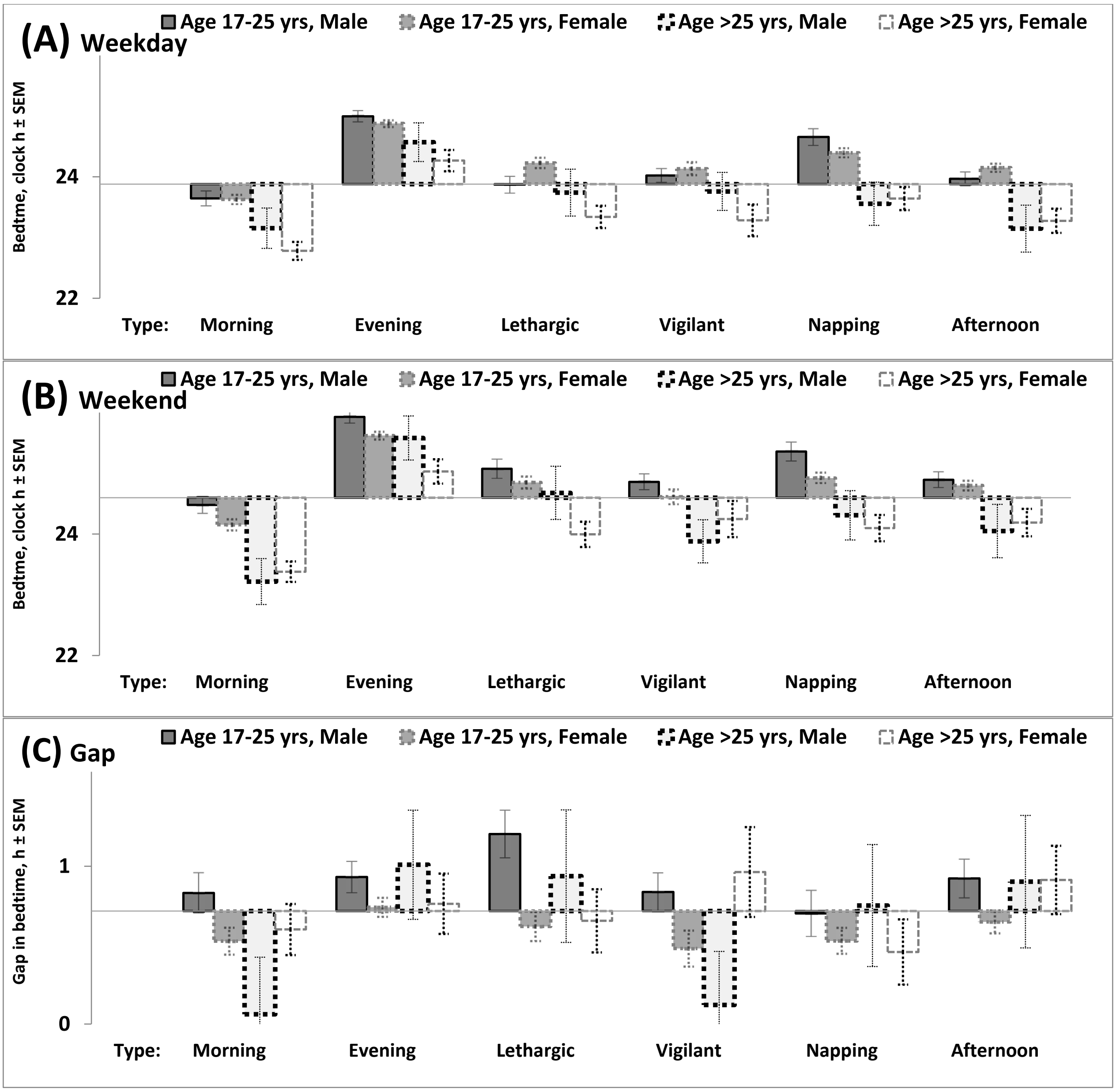

(A) Weekday
Age 17-25 yrs, Male
Age 17-25 yrs, Female
Age >25 yrs, Male
Age >25 yrs, Female
Bedtme, clock h ± SEM
24
22
Type:
Morning
Evening
Lethargic
Vigilant
Napping
Afternoon
(B) Weekend
Bedtme, clock h ± SEM
24
22
(C) Gap
Gap in bedtime, h ± SEM
1
0

Figure S3

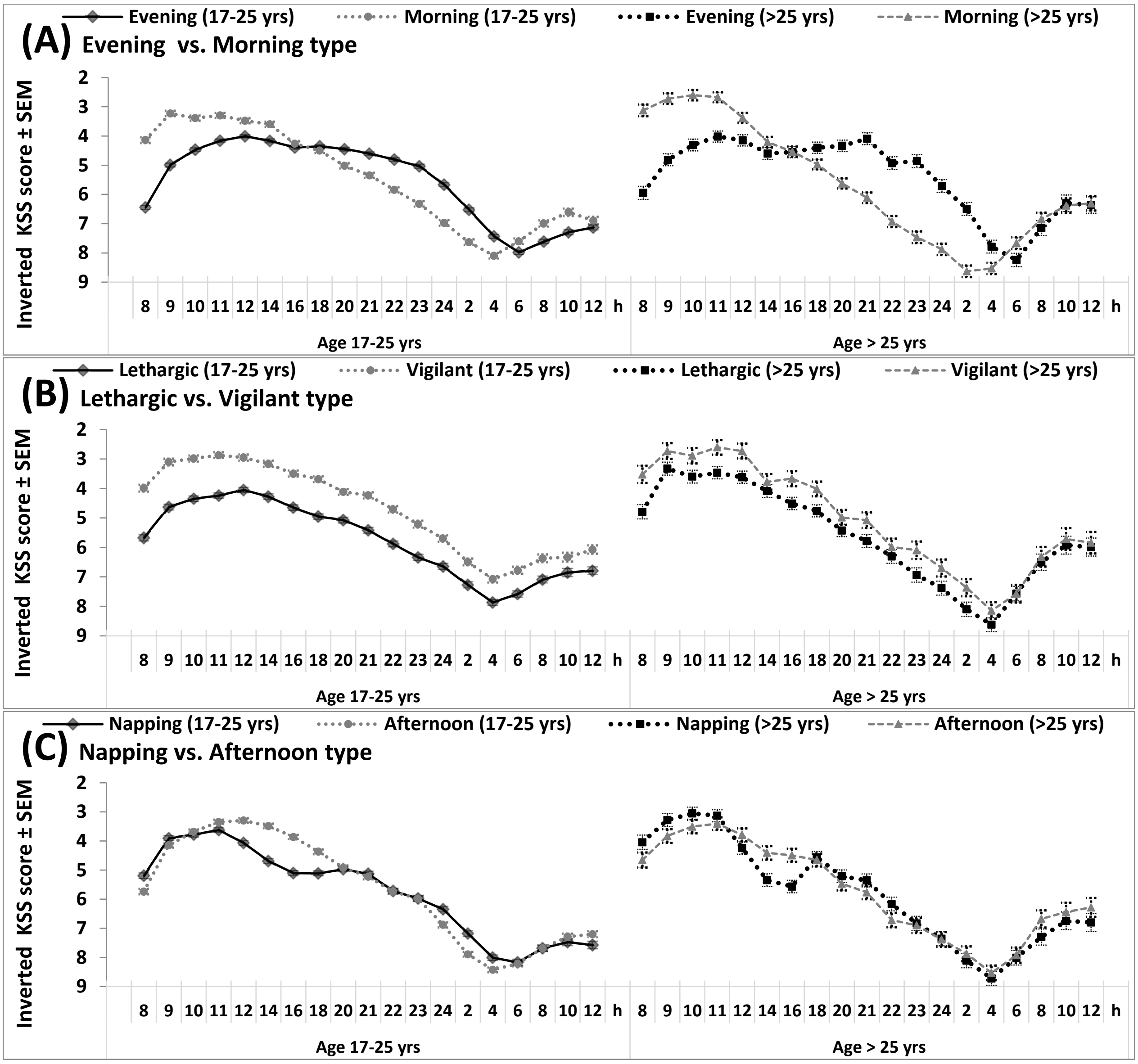

Evening (17-25 yrs)
Morning (17-25 yrs)
Evening (>25 yrs)
Morning (>25 yrs)
(A) Evening vs. Morning type
Inverted KSS score ± SEM
8 9 10 11 12 14 16 18 20 21 22 23 24 2 4 6 8 10 12 h
Age 17-25 yrs
Age > 25 yrs
Lethargic (17-25 yrs)
Vigilant (17-25 yrs)
Lethargic (>25 yrs)
Vigilant (>25 yrs)
(B) Lethargic vs. Vigilant type
Napping (17-25 yrs)
Afternoon (17-25 yrs)
Napping (>25 yrs)
Afternoon (>25 yrs)
(C) Napping vs. Afternoon type

Figure S4

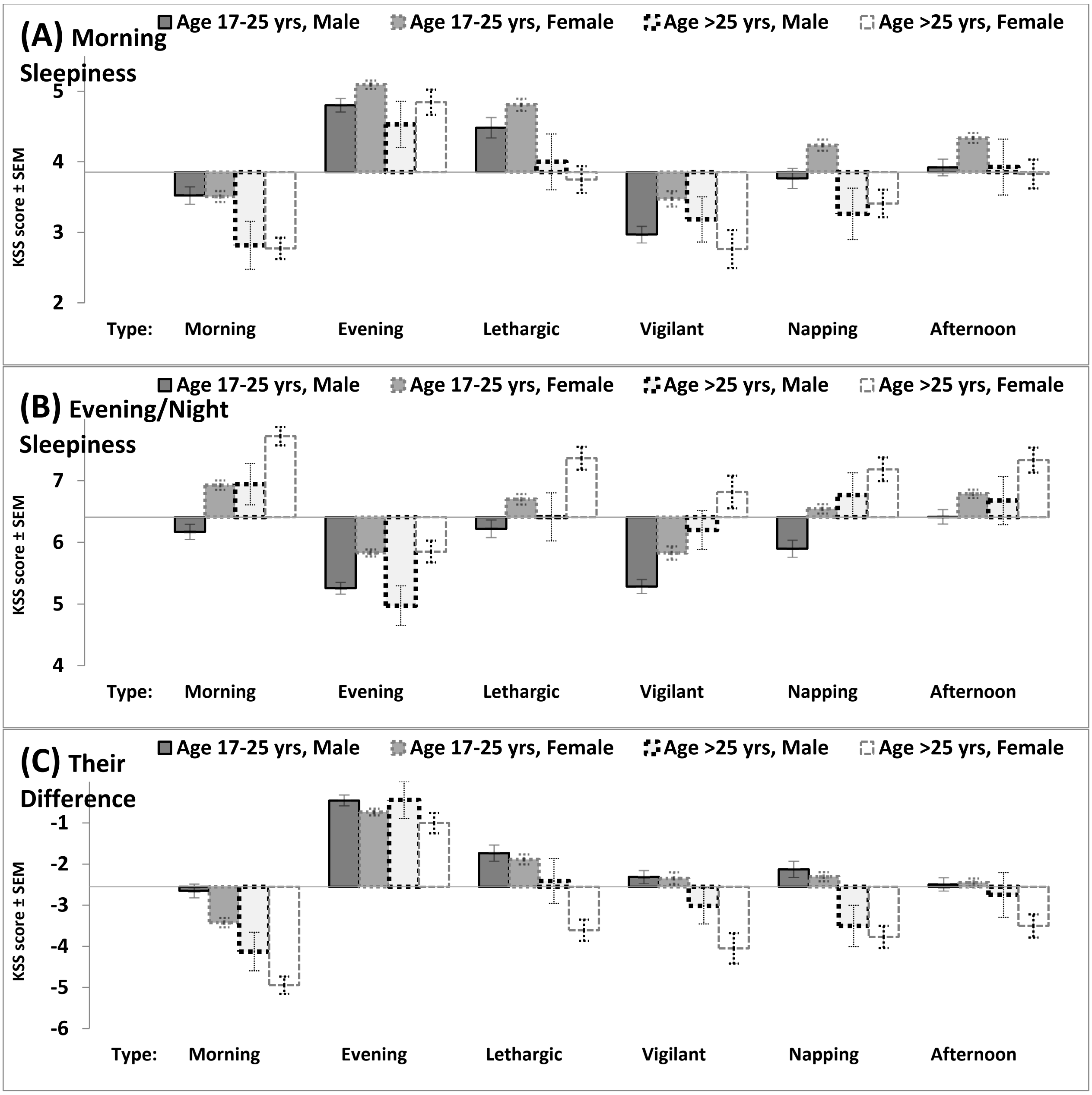
(A) Morning Sleepiness
(B) Evening/Night Sleepiness
(C) Their Difference
Age 17-25 yrs, Male
Age 17-25 yrs, Female
Age >25 yrs, Male
Age >25 yrs, Female
KSS score ± SEM
Type:
Morning
Evening
Lethargic
Vigilant
Napping
Afternoon
5
4
3
2
7
6
5
4
-1
-2
-3
-4
-5
-6

Figure S5

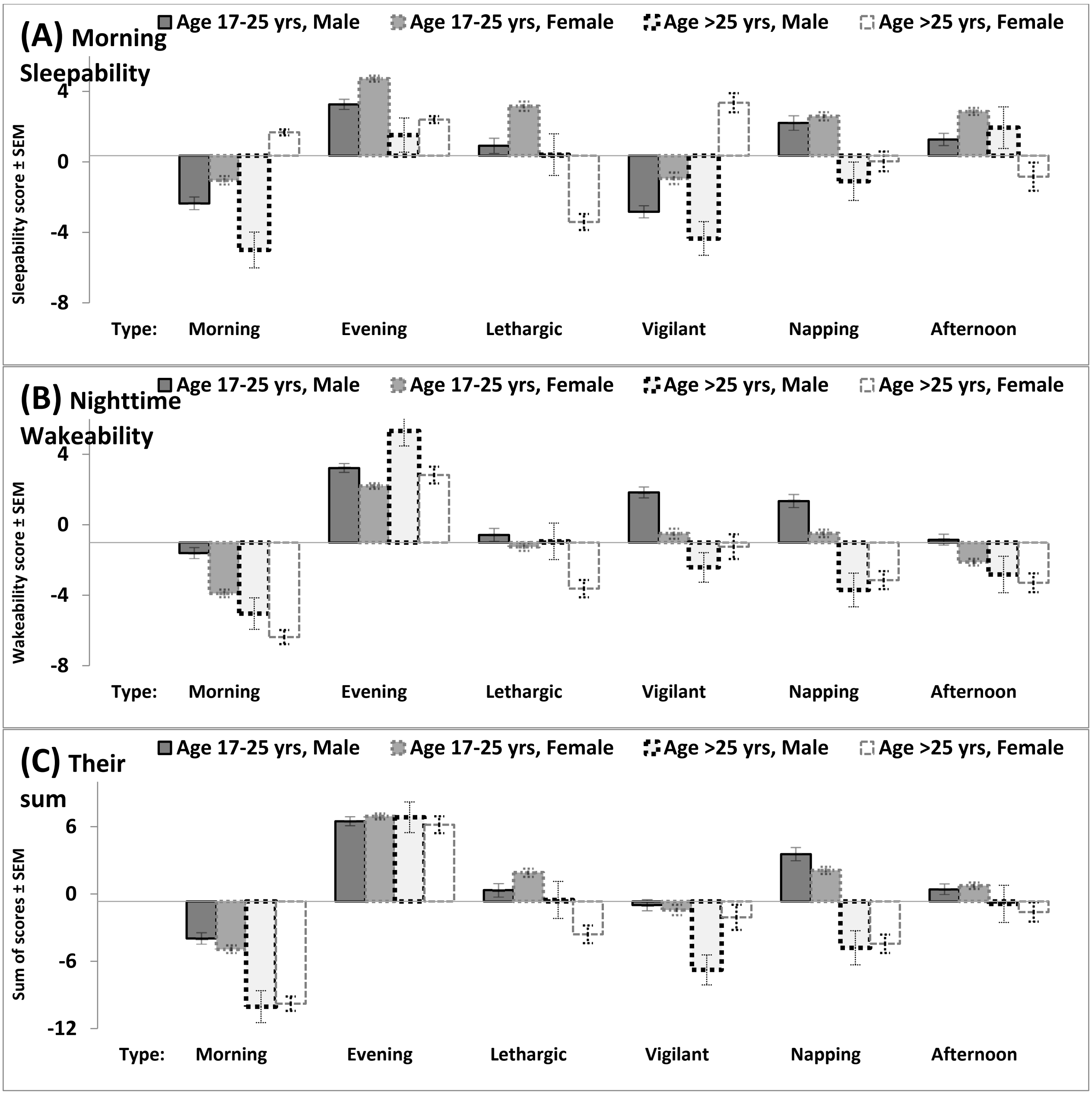
(A) Morning Sleepability
Age 17-25 yrs, Male
Age 17-25 yrs, Female
Age >25 yrs, Male
Age >25 yrs, Female
Sleepability score ± SEM
4
0
-4
-8
Type:
Morning
Evening
Lethargic
Vigilant
Napping
Afternoon
(B) Nighttime Wakeability
Wakeability score ± SEM
(C) Their sum
Sum of scores ± SEM
6
0
-6
-12

Figure S6

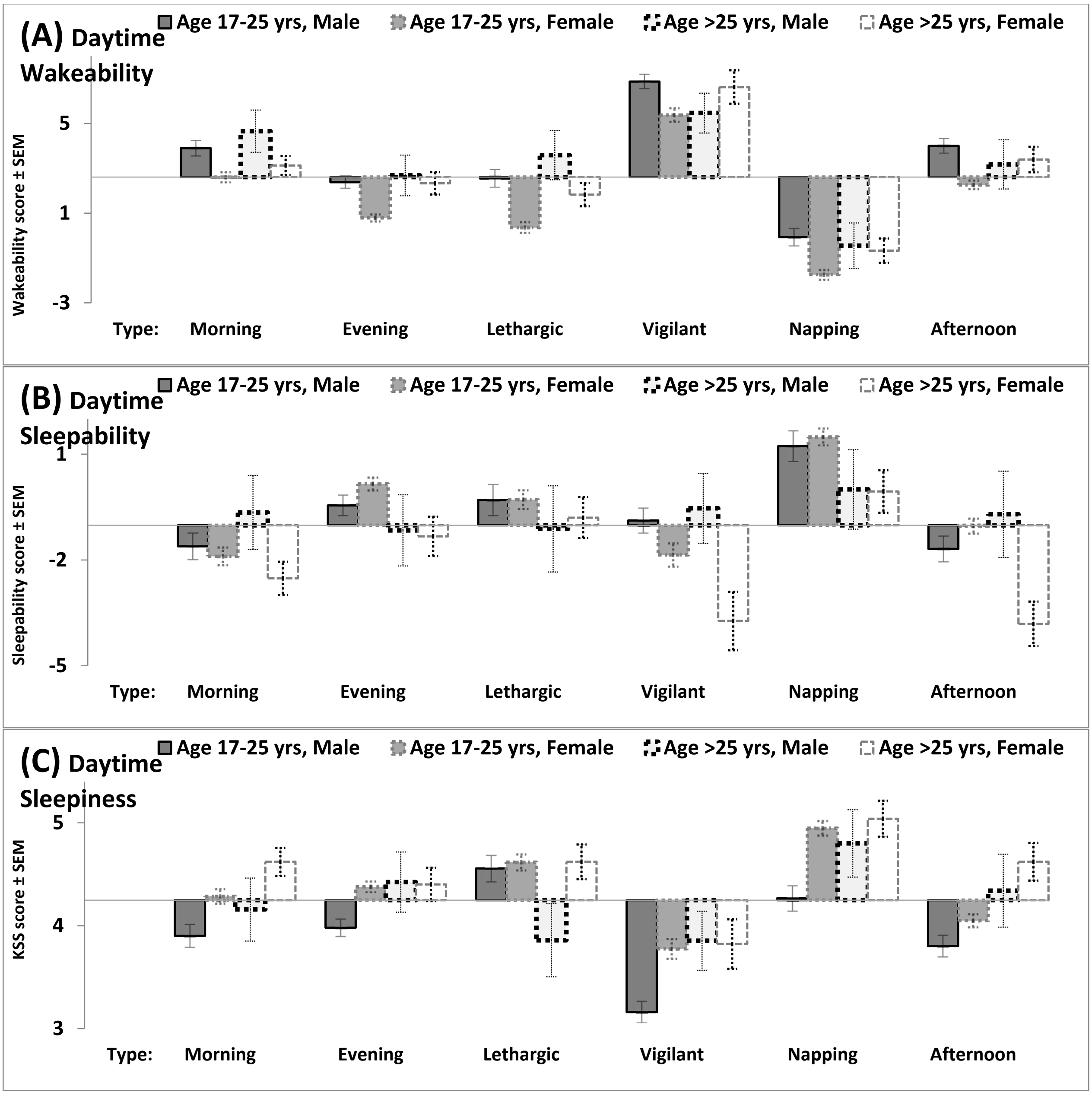

Age 17-25 yrs, Male
Age 17-25 yrs, Female
Age >25 yrs, Male
Age >25 yrs, Female
(A) Daytime Wakeability
Wakeability score ± SEM
5
1
-3
Type:
Morning
Evening
Lethargic
Vigilant
Napping
Afternoon
(B) Daytime Sleepability
Sleepability score ± SEM
1
-2
-5
(C) Daytime Sleepiness
KSS score ± SEM
5
4
3

Figure S7
